# Activatable G-quadruplex based catalases for signal transduction in biosensing

**DOI:** 10.1093/nar/gkad031

**Published:** 2023-02-02

**Authors:** Elzbieta E Iwaniuk, Thuwebat Adebayo, Seth Coleman, Caitlin G Villaros, Irina V Nesterova

**Affiliations:** Department of Chemistry and Biochemistry, Northern Illinois University, DeKalb, IL 60115, USA; Department of Chemistry and Biochemistry, Northern Illinois University, DeKalb, IL 60115, USA; Department of Chemistry and Biochemistry, Northern Illinois University, DeKalb, IL 60115, USA; Department of Chemistry and Biochemistry, Northern Illinois University, DeKalb, IL 60115, USA; Department of Chemistry and Biochemistry, Northern Illinois University, DeKalb, IL 60115, USA

## Abstract

Discovery of oxidative catalysis with G-quadruplex•hemin constructs prompted a range of exciting developments in the field of biosensor design. Thus, G-quadruplex based DNAzymes with peroxidase activity found a niche as signal transduction modules in a wide range of analytical applications. The ability of nucleic acid scaffolds to recognise a variety of practically meaningful markers and to translate the recognition events into conformational changes powers numerous sensor design possibilities. In this work, we establish a catalase activity of G-quadruplex•hemin scaffolds. Catalase activated hydrogen peroxide decomposition generates molecular oxygen that forms bubbles. Observation of bubbles is a truly equipment free signal readout platform that is highly desirable in limited resources or do-it-yourself environments. We take a preliminary insight into a G-quadruplex structure—folding topology—catalase activity correlation and establish efficient operating conditions. Further, we demonstrate the platform's potential as a signal transduction modality for reporting on biomolecular recognition using an oligonucleotide as a proof—of—concept target. Ultimately, activatable catalases based on G-quadruplex•hemin scaffolds promise to become valuable contributors towards accessible molecular diagnostics applications.

## INTRODUCTION

Over the past decades, artificial DNA scaffolds with enzymatic activity (DNAzymes) emerged as powerful signal transduction tools in chemical and biochemical analysis (1,2). Intrinsically stable, non-toxic, and affordable, small nucleic acid scaffolds align with requirements set forth by the World Health Organization (WHO) to operate under limited resources environments (so called ASSURED (Affordable, Sensitive, Specific, User-Friendly, Robust and rapid, Equipment free, Deliverable) criteria (3–6)).

A class of DNAzymes, G-quadruplexes•hemin constructs that mimic protein peroxidases introduced in 1998 by Dipankar Sen *et al.* (7) has advanced into the chemical/biochemical analysis field as translators of target recognition events into separation free readouts. The scaffolds are compatible with a range of detection platforms (e.g. optical (8,9) and electrochemical (10)) including truly equipment-free colorimetric (7,11,12) and liquid-to-gel transition readouts (13). The equipment-free transduction of molecular recognition events is an essential requirement for design of ASSURED-compliant diagnostic devices. However, an interpretation of colour intensity and hue is contingent on lighting conditions and/or on the colour vision proficiency of the observer. Liquid-to-gel transition set up (13) employs acrylamide that is highly toxic and, therefore, is problematic to handle by end-users. Therefore, alternative equipment-free signal readout platforms are still actively searched after.

In this work, we extend the reach of G-quadruplex•hemin scaffolds as signal transduction tools and build up their utility as activatable catalases. Protein catalases catalyse hydrogen peroxide decomposition (2 H_2_O_2_ → 2 H_2_O + O_2_ (*g*)). The reaction produces molecular oxygen that can form bubbles. Observing bubbles is a straightforward and easy-to-interpret route towards instrument-free visualization of a readout (14) that does not require a scientific background, equipped lab, colour vision proficiency and, therefore, can easily expand towards wide scale applications suitable for everyone ages 2 and up.

**Scheme 1.**
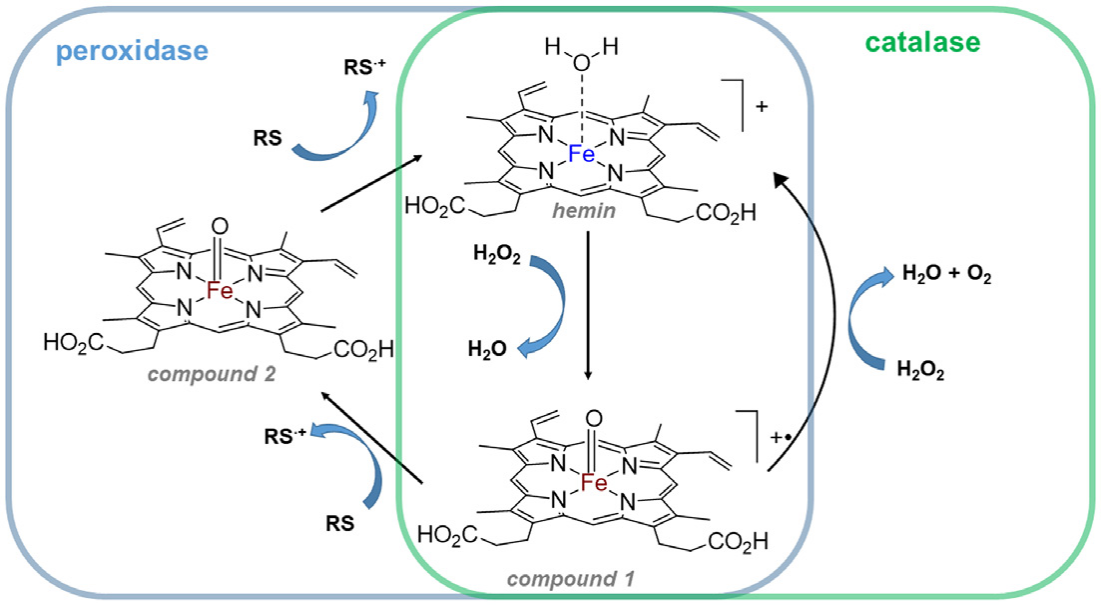
Simplified catalytic cycles of peroxidases and catalases. ‘RS’ = reducing substrate. Iron (III) is blue, iron (IV) is brown.

With no prior knowledge of a catalase activity by nucleic acids scaffolds, we hypothesize that heme peroxidase mimicking G-quadruplex•hemin constructs may function as catalases. Indeed, in protein world, both heme-peroxidases and catalases belong to the same class of metalloenzymes that operate off heme's catalytic activity and their catalytic cycles are similar (7,15–21) (simplified catalytic cycle is included in Scheme 1). Thus, a critical step in both enzymatic pathways is the formation of Compound I (Scheme 1, process 1). In case of catalases, Compound I reacts with a second molecule of H_2_O_2_ yielding O_2_, H_2_O, and the regenerated enzyme (process 2) (19). In peroxidases, Compound I reacts with a reducing substrate (RS) to form Compound II (process 3) followed by regenerating resting enzyme (process 4) (15). Recent experimental proofs (21) and molecular dynamic simulations (19) demonstrating the possibility of a direct (i.e. without participation of a protein frame) transfer of two hydrogen atoms from H_2_O_2_ to the oxoferryl group (process 2) do support our hypothesis. Moreover, recently reported electrochemical measurements prove G-quadruplex-hemin ability to catalyse H_2_O_2_ reduction to water (22,23). Additionally, notoriously promiscuous catalytic behaviour of G-quadruplex DNAzymes (i.e. activity as NADH oxidases (24), NADH peroxidases (24), chelatases (25,26), catalysis of an oxidation of thiols to disulphides (27,28)) gives our hypothesis a further reassurance.

In this work, we establish the catalase activity of G-quadruplex•hemin scaffolds and demonstrate its utility as a signal transduction platform in biomolecular recognition events on a proof-of-concept example of an oligonucleotide target.

## MATERIALS AND METHODS

All the chemical reagents were obtained from established commercial suppliers (i.e. Sigma-Aldrich (St. Louis, MO), Fisher Scientific (Pittsburg, PA) etc.) unless specifically mentioned below. Nuclease-free water used for preparation of DNA solutions was obtained from IDT (Coralville, IA). 10 × PBS Buffer (pH 7.4) was purchased from Thermo Fisher Scientific (Waltham, MA); UltraPure 1M Tris–HCl (pH 7.5) Buffer was purchased from Invitrogen (Grand Island, NY) Single stranded DNAs were obtained from IDT (Coralville, IA) and reconstituted with nuclease-free water. Oligonucleotide sequences are included in Table S1. pH-meter calibration buffers (4.00 ± 0.01, 7.00 ± 0.01 and 10.00 ± 0.02) were purchased from Fisher Scientific. DNA TriDye Ultra Low Range DNA Ladder was purchased from New England Biolabs (Ipswich, MA). SYBR Gold staining dye was purchased from Thermo Fisher Scientific. Acrylamide:bis-Acrylamide (29:1) solution for PAGE was obtained from BioRad (Hercules, CA). Hydrogen Peroxide (30%) was purchased from Fisher Scientific. Hemin was purchased from Thermo Fisher Scientific. TRIS buffers supplemented with NaCl and KCl were prepared in our laboratory.

### G-quadruplex preparation

G-quadruplexes were prepared by denaturing an approximately 150 μl of a 10–50 μM oligonucleotide solution in an appropriate buffer at 95.0°C for 5.0 min followed by overnight cooling to room temperature. The actual concentration of G-quadruplexes was established post-equilibration using 260 nm absorption values obtained by UV–vis and extinction coefficients provided by IDT.

### UV–vis measurements

Cary 4000 UV-Vis spectrophotometer (Agilent Technologies, Santa Clara, CA) was used for all UV measurements. Typically, measurements were performed on 800-μl solution aliquots using a 10 mm optical path quartz cuvette. For establishing oligonucleotide concentrations, ∼1 μM dilutions were analyzed.

### Measuring pH

ThermoOrion pH-meter (Model 420) was used throughout the studies. The pH meter was calibrated against fresh aliquots of pH calibration buffers before each use.

### Circular dichroism (CD) spectrometry (Supplementary Figure S1)

CD spectra were collected on AVIV Model 215 Circular Dichroism Spectrometer (Aviv Instruments Inc., Lakewood, NJ) from 200 to 320 nm. Solutions containing equilibrated G-quadruplexes (prepared as described in G-quadruplex preparation subsection above) were diluted to ∼5 μM with PBS buffer at pH of 7.5. Quartz cuvettes with a 10-mm light path were used for all CD measurements. CD spectra for split structures were recorded in the presence of the equivalent amount of target T1. To correct for a signal from DNA duplex in target/G-quadruplex constructs, we subtracted the control ‘duplex’ (target T1 + 9:3 split T control L + 9:3 split T control R) spectrum from the corresponding split G-quadruplexes + target T1 spectra.

The instrument output (θ, mdeg) was converted into molar ellipticity (per strand, (Δϵ, mdeg·M^−1^·cm^−1^) using the equation Δϵ = θ/(*M* × *L* × 32 980), where *M* is oligonucleotide strand concentration (mol/L) and *L* is optical path length (cm).

### Hemin solutions

A 2.8 mM hemin's stock was prepared by dissolving 9 mg of hemin in 5 ml of DMSO. A 250 μM working solution was diluted from the stock in DMSO.

### G-quadruplex–hemin binding (Figure 1, Supplementary Figure S2)

Hemin stock (in DMSO) was spiked into PBS (pH 7.5) and the initial (*t* = 0) time point was recorded. Next, equilibrated GI–GVII (prepared as discussed in ‘G-quadruplex preparation’, *vide supra*) were added to hemin. The final concentration of hemin was 1 μM and of an oligonucleotide—500 nM. Absorption spectra were recorded from 300 to 500 nm every 5 min for 60–75 min.

### Catalase activity of full G-quarduplexes GI–GVII (Figure 2, Supplementary Figure S3)

**Figure 1.**
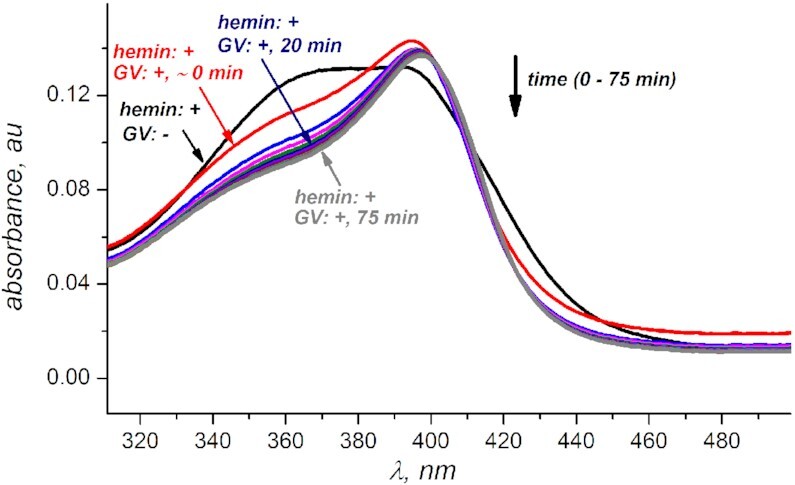
Bathochromic shift and increase in intensity of hemin's Soret band indicates hemin's de-aggregation upon interacting with G-quadruplex V. Data for other quadruplexes indicate to a similar trend (Supplementary Figure S2). Hemin concentration is 1 μM, GV concentration is 500 μM in PBS buffer at pH 7.50.

For naked eye observations, we mixed 40 μl of equilibrated 10–50 μM G-quadruplex solution (described above in G-quadruplex preparation) and 8 μl of a hemin working solution (250 μM in DMSO) in a 20-ml glass scintillation vial. The mixture was equilibrated for 20 minutes at ambient conditions to let hemin interact with G-quadruplex. Then 1952 μl of 30% hydrogen peroxide was added (bringing total H_2_O_2_ concentration to 29.3%); and bubble observations were started. Addition of hydrogen peroxide is a ‘0 min’ point in Figure 1 and Supplementary Figure S3. After indicated time points, bubbles on the surface of solution were observed either with a naked eye; or some images (view from above) were taken with a cell phone camera (iPhone XR). An equivalent volume of buffer instead of G-quadruplex solution was used in control samples.

### Gel electrophoresis (Supplementary Figure S4)

Gels for polyacrylamide gel electrophoresis (PAGE) were prepared in our laboratory. Typically, 4 μl of ∼4 μM oligonucleotide solutions mixed with glycerol (to yield ∼17%) were separated in 0.5× TBE running buffer on a 20% gel at 150 V for ∼2 h at 4°C using a VWR (Radnor, PA) vertical PAGE system and a power source. Gels were stained with SYBR Gold (3 μl dye in 200 ml of 0.5× TBE buffer) for 20 min and imaged with Bio-Rad EZ Gel Doc imager (Hercules, CA). Single stranded oligonucleotides with no G-quadruplex forming sequences (21-, 22- and 24-nt) were injected as unfolded controls.

### Thermal folding studies (Supplementary Figure S5)

Sequences GI–GVII (at 500 nM) in PBS (pH 7.50) were denatured at 80°C for 30 min in cuvette compartment of UV–vis spectrometer followed by cooling to 1–10°C at a 0.2°C/min rate. An Absorbance at 295 indicative of G-quadruplex folding state was measured each 1°C.

### Catalase activity upon target detection (Figure 3, Supplementary Figure S6) and quantitative analysis (Supplementary Figure S7)

Split G-quadruplexes were prepared by denaturing of ∼50 μM solutions (with exact concentrations established by UV–vis) of a left arm strand, a right arm strand, and a target in a PBS buffer (pH 7.5) at 95.0°C for 5.0 min, slowly cooling down to room temperature, and equilibrating for additional 10 h. Further sample preparation proceeded as described above for full G-quadruplexes GI–GVII but with the split G-quadruplex/target hybrids. For controls, the samples with equivalent amounts of left and right arm strands but no target were equilibrated and treated in an identical fashion.

For microscopic observations (Figure 3), images were taken with BZ-X800 Analyzer (Keyance, Japan). The solutions were prepared in a 96-Well Plate and images were taken in Bright Field with a 4× objective. The reaction volumes were scaled down to yield the same concentrations and reagent ratios as indicated above in a total volume of 300 μl. Sample preparation/observation timelines were equivalent to those used for naked eye observations.

## RESULTS AND DISCUSSION

### G-quadruplex design and environmental conditions

To design activatable catalases, we focus on G-quadruplexes with at least three G-quartets because those are known to form tight complexes with hemin (29–31). Since folding topology may influence catalytic properties of G-quadruplexes (29), as a starting point, we choose structures with enough flexibility to fold either into a parallel or an antiparallel conformation. Thus, our core oligonucleotide GI with a sequence (d(GGGTTA)_3_GGG) is a human telomere repeat with three G-quartets reported to form a parallel or antiparallel structure under various conditions (32) and to enhance a peroxidase activity of hemin (33). Considering similarities in catalytic cycles of peroxidases and catalases (Scheme 1, *vide supra* for discussion), we expect the core quadruplex to mimic a catalase. Further, we deliberately introduce some incremental modifications into the core's loops and added overhangs to generate four other three-G-quartet sequences GII–GV. As controls, we designed two G-quadruplex sequences (GVI and GVII) that cannot form ‘canonical’ three-G-quartet structures essential for hemin activation in DNAzymes (29–31). All sequences are included in Table S1.

To select buffer conditions, we take into account that: (i) the hemin-catalyzed hydrogen peroxide decomposition is the most efficient at pHs around 7–8 (34); (ii) G-quadruplex folding requires a presence of Na^+^ or K^+^ ions (35,36); (iii) G-quadruplex/hemin DNAzymes show higher peroxidase activity at pH 7–8 (37,38); (iv) optimal activity of catalases is observed over pH range of 7–11 (39). Therefore, for initial evaluations we selected three buffering systems that satisfy the criteria: TRIS + 50 mM KCl (pH 8.0), Tris + 50 mM NaCl (pH 8.0) and PBS (pH 7.5).

### Catalase activity of G-quadruplexes

#### Hemin binding

To ensure that G-quadruplexes bind hemin (a condition for the activation of G-quadruplex DNAzymes (40)), we monitored the Soret band on the hemin adsorption spectra. Increase in intensity and bathochromic shift observed (Figure 1, Supplementary Figure S2) is consistent with hemin binding a G-quadruplex scaffold (38,41–42). We found no significant changes to the Soret band beyond the first 20 minutes of hemin/G-quadruplex interaction (for all quadruplexes except GVII that appears to be binding hemin much slower). Therefore, we incorporate at least 20 min G-quadruplex•hemin equilibration time before introducing hydrogen peroxide into all experimental conditions discussed below.

#### Catalase activity

**Figure 2.**
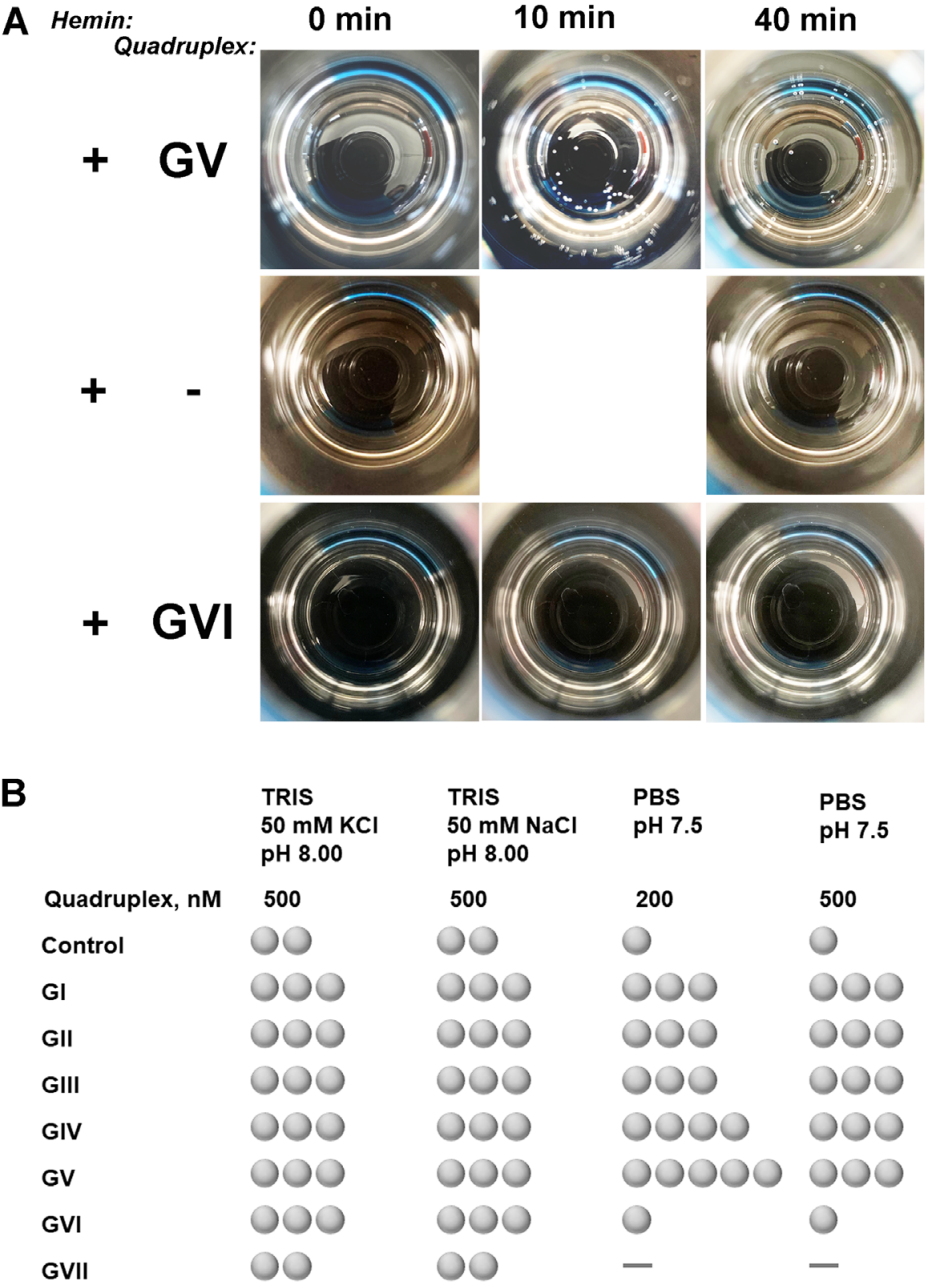
Representative images (**A**) and summary (**B**) of bubbles formation in presence of quadruplexes GI–GVII. Samples were prepared in PBS (pH 7.5) and contained equilibrated G-quadruplexes at indicated levels, hemin at 1 μM, and 29.3% of hydrogen peroxide. Control consisted of all the components except a G-quadruplex. Images in (A) are views from above on 20-ml scintillation vials taken with a cell phone camera. Observations in (B) are taken 30 min after adding hydrogen peroxide. One ‘bubble’ symbol in (B) corresponds to 1–3 bubbles observed, two to 4–8 bubbles, three to 8–12 bubbles, four to 12–18 bubbles, five to almost all the surface area covered (more than 18), dash corresponds to zero (0) bubbles.

To evaluate the catalase activity, we exposed GI–GVII to hydrogen peroxide in the presence of hemin in all three buffering systems (*vide supra*). To ensure hemin binding, we equilibrated G-quadruplex/hemin mixture for 20 min before adding H_2_O_2_. Upon preliminary optimization of H_2_O_2_ concentration (over a 3–27.3% range), G-quadruplex concentration (over a 100–2000 nM range), hemin : G-quadruplex ratio (over a 5:1 to 1:2 range), aqueous/DMSO ratio (over a 0.1–1% range), we came up with optimal conditions consisting of 200–500 nM of a G-quadruplex oligonucleotide, below 0.5% of DMSO, 2:1 to 5:1 of hemin/G-quadruplex ratio, and ∼29% of H_2_O_2_ (final concentration after all the components are mixed with a conventionally available solution of 30% H_2_O_2_). Our observations taken in the optimized conditions (Figures 2 and Supplementary Figure S3) indicate: (i) control experiments (in absence of any G-quadruplex) in both versions of TRIS buffer demonstrate substantially higher background bubbling levels compared to PBS; (ii) quadruplexes GI–GV produce bubbles in amounts exceeding corresponding controls under all buffering conditions evaluated; (iii) GVII (in all conditions) and GVI (in PBS) do not produce bubbles in amounts that observably exceed control levels. Those observations clearly indicate that quadruplexes GI–GV noticeably enhance the catalase activity of hemin with the best signal to background ratio obseved in PBS buffer at pH 7.5.

#### Correlation between the catalase activity and G-quadruplex folding state and topology

Rational development of G-quadruplex based catalases requires understanding G-quadruplex structure–activity link. To establish the link, we evaluate the folding stability and folding topology of GI–GVII under the selected conditions.

**Figure 3.**
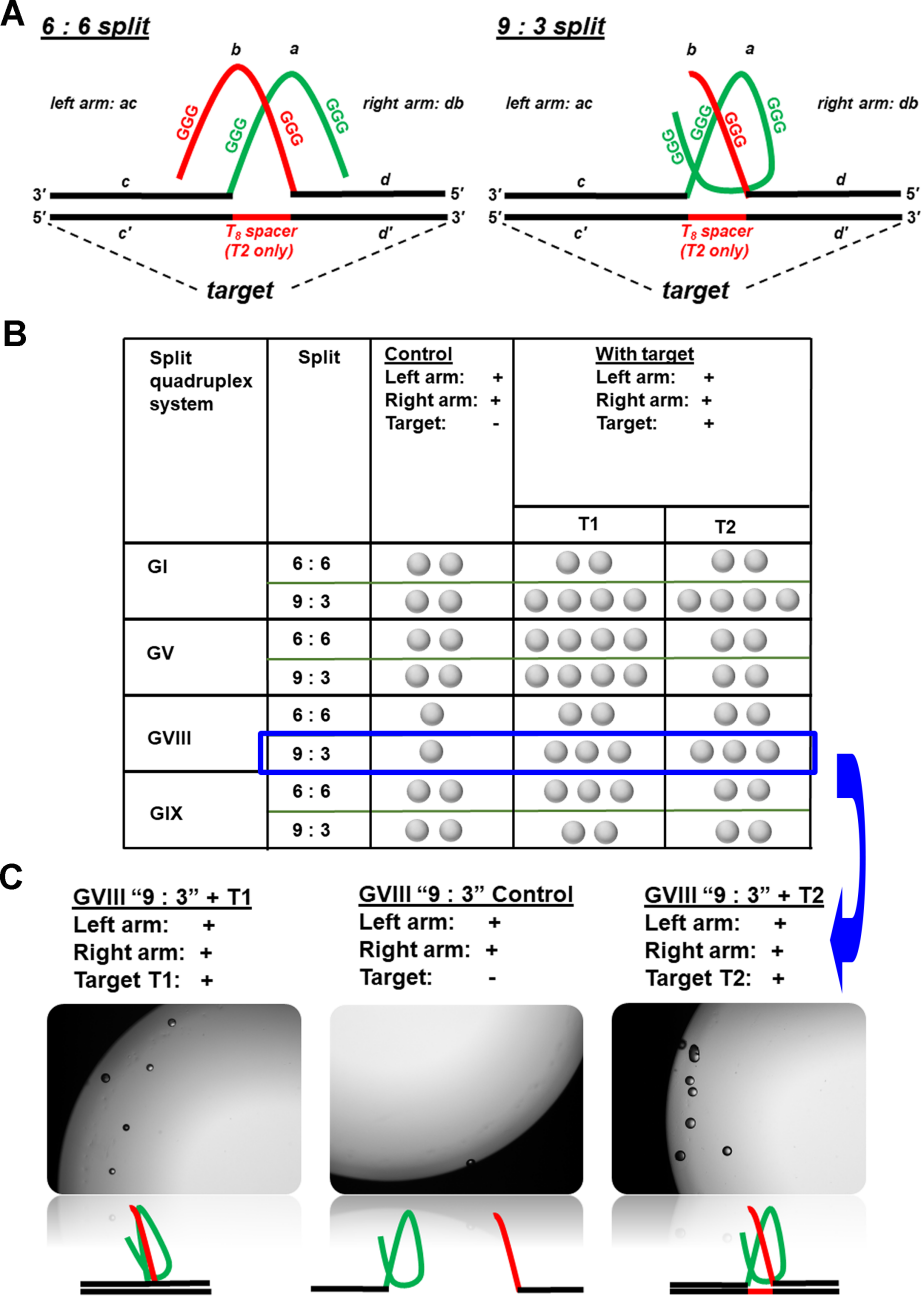
(**A**) Activation of catalase activity of quadruplexes GVIII and GIX in presence of an oligonucleotide target requires splitting guanine-rich sequences in two parts. We evaluate two different split patterns: a symmetric (6:6) and an asymmetric (9:3). (**B**) Results over different split and target configurations. Each sample in PBS buffer (pH 7.50) consisted of two quadruplex ‘arms’ (R and L) and target (all at 500 nM concentrations), hemin at 1 μM, and hydrogen peroxide at 29.3%. Control consisted of all the components except an oligonucleotide target. Observations were taken 30 min after adding hydrogen peroxide. One ‘bubble’ symbol in the table correspond to 1–3 bubbles observed, two to 4–8 bubbles, three to 8–12 bubbles. (**C**) Representative image of response to the target T1 (left) and T2 (right) against a control (no target, centre) taken with 4× objective using Keyance microscope. The samples were prepared in a 96-well plate. The total sample volume was 300 μl. Additionally, conventional cell phone images for GV ‘9:3’ splits are included in Figure S6.

Evaluation of thermal folding profiles (Supplementary Figure S5) indicate mostly folded conformation of GI and GV over ambient temperature range (20–25°C), a mixture of folded and unfolded confirmations for GII–GIV and GVI, and mostly unfolded state of GVII. On non-denaturing gel electropherograms (Supplementary Figure S4), bands for GI, GII, GIV, GV and GVII migrate father than corresponding non-structured strands of identical lengths. Migration of GIII and GVI does not differ from controls. Some variances observed between gels and thermal profiles (e.g. folded state for GVII is evident on gel but no folding appears on thermal profiles or no folding of GIII and GVI on gel but partial folding follows from the corresponding thermal profiles) may originate from low temperature of gel separations (4°C) and/or the presence of glycerol in samples. Importantly, both experiments point towards consistently stable folded state of GV, a sequence that shows the highest catalase enhancement in PBS (Figure 3).

To get a preliminary insight into a correlation between the folding topology and catalase activity, we interrogate CD spectra of all the G-quadruplexes (Supplementary Figure S1). Importantly, the CD spectra for GI, GII, GIV and GV are consistent with spectra reported for telomeric G-quadrplex sequences in phosphate buffer: maximum between 290 and 295 nm, minimum around 265 nm, and maximum around 240–250 nm (32). The features agree well with antiparallel folding topology (43,44). The quadruplexes do not indicate substantial folded confirmation either on gel (GIII and GVI) or on thermal folding (GVII) have features indicating the presence of single stranded species (higher signal or even maximum around 260 nm). The CD spectra of GIII and GVII do not correlate with any the three clearly defined topological systems (parallel, antiparallel or hybrid/3 + 1) (43,44) indicating either to a mixture of conformations or a substantial fraction of nonstructured strands;); CD spectrum of GVI has some features consistent with hybrid topology.

Overall, on example of sequence GV, we conclude that the stability at ambient conditions and antiparallel folding topology correlate with catalase activity enhancement. However, considering the complexity in G-quadruplexes’ folding topology and its contingency on oligonucleotide sequence and ionic environments (45), we are currently designing more thorough studies to understand the structure/property correlation.

### Activatable G-quadruplex catalases

To enable a target-triggered bubble production, we engineer a target-templated G-quadruplex assembly. We split G-quadruplexes into two fragments with a vision that the fragments that are separated from each other are unlikely to form a quadruplex. When deliberately brought into proximity, the fragments assemble into a quadruplex structure. We make the quadruplex assembly contingent on the presence of a target oligonucleotide strand. For the proof-of-concept demonstrations, we derive a target from a rat prolactin promoter sequence (used as a model in (46) and our prior publications (47,48)). We split G-quadruplexes into left (L) and right R ‘arms’ (Figure 3, domains *a* and *b*) and extend the ‘arms’ with target recognition domains (*c* and *d*) that are complementary to fragments *c’* and *d’* on a target. Therefore, in the presence of target, c/c’ and d/d’ hybridization brings domains *a* and *b* in a close proximity triggering a G-quadruplex assembly. It is known that split design may influence the effectiveness G-quadruplex reassembly (49). Therefore, as a starting point, we evaluate different split types, symmetric (6:6) and assymetric (9:3). In the symmetric (6:6) split both arms contain two three - guanine stretches while in the assymetric split, the right arm contains one three - guanine stretch while the left arm contains three such stretches (Figure 3A). The type of assymetric split (9:3) has been demonstrated as beneficial against one with one three – guanine stretch on left arm and three stretches on the right arm (50). To follow the design considerations, we obtained four split G-quadruplex systems: two based on GI and GV (as most stable in ambient conditions) and two other systems, GVIII and GIX (Table S1, Figure 3A). We evaluate two target conformations: target T1 with domains *c’* and *d’* adjacent to each other and target T2 with domains *c’* and *d’* separated by a 8-nucleotide ‘spacer’.

Evaluation of the activatable setups shows that asymmetric 9:3 splits perform better (GI and GVIII) or similarly (GV) when tested against symmetrical 6:6 splits (Figures 3 and Supplementary Figure S6). The finding is consistent with previous reports that asymmetrically split G-quadruplexes demonstrate higher peroxidase activity and form tighter complexes with protoporphyrin IX (a compound structurally similar to hemin) (49–52). Also, we did not observe a significant difference in target configuration except for GV demonstrating clear preference for T1 with adjacent hybridization domains over T2 with a spacer between hybridization arms spaced.

### Activatable catalases, their limitations, and future studies

The G-quadruplex/hemin based activatable catalases promise to evolve into a new equipment-free detection platform. Currently, we established their utility at 200 nM concentrations. While many nucleic acid biomarkers are relevant at lower concentrations (53), recent progress in isothermal amplification techniques (54) (including ones that are ASSURED-compliant) will enable effective equipment-free detection of many relevant biomolecular targets.

Further, we hypothesize that lower detection levels can be achieved even without upfront amplification. Indeed, we demonstrated a link between an increased stability of G-quadruplexes (on an example of GV) and catalase activation. Considering that GII–GIV appear to be partially folded at ambient conditions (based on thermal profiles in Figure S5) but still yield a noticeable signal to background ratios (Figure 2), we assume that lower concentrations of quadruplexes may be sufficient (only fractions of GII–GIV are folded). Therefore, we expect that stable quadruplexes may be effective at much lower concentration.

Moreover, we are very enthusiastic about extending the catalase system into a field of quantitative analysis. The target-assembly activatable system (split) is compatible with a stoichiometric approach for quantitative analysis of biomolecules we developed earlier (47,48). We have preliminary demonstrated that the approach enables naked eye quantitative analysis of a target T1 at 500 nM level (Supplementary Figure S7). Further refinements of the detection scheme are currently under way in our lab.

## CONCLUSIONS

In conclusion, we demonstrate that G-quadruplex•hemin constructs are capable of mimicking protein catalases and, as such, to activate hydrogen peroxide decomposition. The reaction produces molecular oxygen that, when released in form of bubbles, serves as a promising visual signal readout platform for molecular recognition events.

We design activatable G-quadruplex•hemin-based catalases that produce an easily observable signal readout in the presence of an oligonucleotide target. However, considering a wide range of target recognition possibilities by nucleic acid scaffolds (55), our work expands the reach of DNAzymes as sensor design platforms (56); we specifically target ASSURED-compliant applications.

Overall, the significance of activatable catalases can extend beyond pure analytical applications into therapeutic area where reactive oxygen species scavengers are in a high demand (57).

We preliminarily establish structure–function correlation for G-quadruplex•hemin catalase mimics and found that enhancement of hemin's catalase activity requires stable G-quadruplex structures with at least three G-quartets. Further, we demonstrate that the catalase activity of G-quadruplex•hemin scaffolds correlate with antiparallel folding topology. However, the work in this direction is to be continued considering that in the world of G-quadruplex•hemin peroxidases parallel folds are the ones that yield the most efficient DNAzymes (40).

## DATA AVAILABILITY

All the data that support this article are included within the main text and Supplementary Information available online.

## Supplementary Material

gkad031_Supplemental_File

## References

[1] Peng H.Y. , Newbigging A.M., Wang Z.X., Tao J., Deng W.C., Le X.C., Zhang H.Q. DNAzyme-mediated assays for amplified detection of nucleic acids and proteins. Anal. Chem. 2018; 90:190–207. 29183114 10.1021/acs.analchem.7b04926

[2] McConnell E.M. , Cozma I., Morrison D., Li Y. Biosensors made of synthetic functional nucleic acids toward better human health. Anal. Chem. 2020; 92:327–344. 31656066 10.1021/acs.analchem.9b04868

[3] Peeling R.W. , Holmes K.K., Mabey D., Ronald A. Rapid tests for sexually transmitted infections (STIs): the way forward. Sex. Transm. Infect. 2006; 82:V1–V6. 17151023 10.1136/sti.2006.024265 PMC2563912

[4] Peeling R.W.W. , Mabey D. Point-of-care tests to reduce the burden of sexually transmitted infections. Lancet Infect. Dis. 2019; 19:570–571. 31036512 10.1016/S1473-3099(18)30783-7

[5] Pai N.P. , Vadnais C., Denkinger C., Engel N., Pai M. Point-of-care testing for infectious diseases: diversity, complexity, and barriers in low- and middle-income countries. PLoS Med. 2012; 9:e1001306. 22973183 10.1371/journal.pmed.1001306 PMC3433407

[6] Smith S. , Korvink J.G., Mager D., Land K. The potential of paper-based diagnostics to meet the ASSURED criteria. RSC Adv. 2018; 8:34012–34034. 35548839 10.1039/c8ra06132g PMC9086909

[7] Travascio P. , Li Y.F., Sen D. DNA-enhanced peroxidase activity of a DNA aptamer-hemin complex. Chem. Biol. 1998; 5:505–517. 9751647 10.1016/s1074-5521(98)90006-0

[8] Liu X.Q. , Freeman R., Golub E., Willner I. Chemiluminescence and chemiluminescence resonance energy transfer (CRET) aptamer sensors using catalytic hemin/G-quadruplexes. ACS Nano. 2011; 5:7648–7655. 21866963 10.1021/nn202799d

[9] Monchaud D. , Teulade-Fichou M.-P. A Hitchhiker's guide to G-quadruplex ligands. Org. Biomol. Chem. 2008; 6:627–636. 18264563 10.1039/b714772b

[10] Chen S. , Liu P., Su K.W., Li X., Qin Z., Xu W., Chen J., Li C.R., Qiu J.F. Electrochemical aptasensor for thrombin using co-catalysis of hemin/G-quadruplex DNAzyme nnd octahedral Cu_2_O-Au nanocomposites for signal amplification. Biosens. Bioelectron. 2018; 99:338–345. 28800505 10.1016/j.bios.2017.08.006

[11] Du Y. , Li B.L., Wang E.K. Fitting” Makes “Sensing” simple: label-free detection strategies based on nucleic acid aptamers. Acc. Chem. Res. 2013; 46:203–213. 23214491 10.1021/ar300011g

[12] Hollenstein M. DNA catalysis: the chemical repertoire of DNAzymes. Molecules. 2015; 20:20777–20804. 26610449 10.3390/molecules201119730 PMC6332124

[13] Fedotova T.A. , Kolpashchikov D.M. Liquid-to-gel transition for visual and tactile detection of biological analytes. Chem. Commun. 2017; 53:12622–12625. 10.1039/c7cc07035g PMC5748337 29082399

[14] Chen H. , Li Z., Zhang L.Z., Sawaya P., Shi J.B., Wang P. Quantitation of femtomolar-level protein biomarkers using a simple microbubbling digital assay and bright-field smartphone imaging. Angew. Chem., Int. Ed. 2019; 58:13922–13928. 10.1002/anie.201906856 PMC7211056 31344297

[15] Marnett L.J. , Kennedy T.A. de Montellano P.R.O. Comparison of the peroxidase activity of hemoproteins and cytochrome P450. Cytochrome P450. 1995; Boston, MA Springer 49–80.

[16] Kosman J. , Juskowiak B. Seitz H. , Stahl F., Walter J.-G. Bioanalytical application of peroxidase-mimicking DNAzymes: status and challenges. Catalytically Active Nucleic Acids. 2020; Cham Springer International Publishing 59–84. 10.1007/10_2017_7 28474157

[17] Stefan L. , Denat F., Monchaud D. Insights into how nucleotide supplements enhance the peroxidase-mimicking DNAzyme activity of the G-quadruplex/hemin system. Nucleic Acids Res. 2012; 40:8759–8772. 22730286 10.1093/nar/gks581 PMC3458538

[18] Jones P. , Dunford H.B. The mechanism of compound I formation eevisited. J. Inorg. Biochem. 2005; 99:2292–2298. 16213024 10.1016/j.jinorgbio.2005.08.009

[19] Alfonso-Prieto M. , Biarnes X., Vidossich P., Rovira C. The molecular mechanism of the catalase reaction. J. Am. Chem. Soc. 2009; 131:11751–11761. 19653683 10.1021/ja9018572

[20] Kato S. , Ueno T., Fukuzumi S., Watanabe Y. Catalase reaction by myoglobin mutants and native catalase - mechanistic investigation by kinetic isotope effect. J. Biol. Chem. 2004; 279:52376–52381. 15347658 10.1074/jbc.M403532200

[21] Watanabe Y. , Nakajima H., Ueno T. Reactivities of oxo and peroxo intermediates studied by hemoprotein mutants. Acc. Chem. Res. 2007; 40:554–562. 17567089 10.1021/ar600046a

[22] Yang Q.L. , Nie Y.J., Zhu X.L., Liu X.J., Li G.X. Study on the electrocatalytic activity of human telomere G-quadruplex-hemin complex and its interaction with small molecular ligands. Electrochim. Acta. 2009; 55:276–280.

[23] Aizen R. , Golub E., Trifonov A., Shimron S., Niazov-Elkan A., Willner I. G-Quadruplex-stimulated optical and electrocatalytic DNA switches. Small. 2015; 11:3654–3658. 25903041 10.1002/smll.201403794

[24] Golub E. , Freeman R., Willner I. A hemin/G-quadruplex acts as an NADH oxidase and NADH peroxidase mimicking DNAzyme. Angew. Chem., Int. Ed. 2011; 50:11710–11714. 10.1002/anie.201103853 22229160

[25] Peng D. , Li Y., Huang Z., Liang R.-P., Qiu J.-D., Liu J. Efficient DNA-catalyzed porphyrin metalation for fluorescent ratiometric Pb2+ detection. Anal. Chem. 2019; 91:11403–11408. 31414597 10.1021/acs.analchem.9b02759

[26] Li Y.F. , Sen D. A catalytic DNA for porphyrin metallation. Nat. Struct. Biol. 1996; 3:743–747. 8784345 10.1038/nsb0996-743

[27] Golub E. , Freeman R., Willner I. Hemin/G-quadruplex-catalyzed aerobic oxidation of thiols to disulfides: application of the process for the development of sensors and aptasensors and for probing acetylcholine esterase activity. Anal. Chem. 2013; 85:12126–12133. 24299064 10.1021/ac403305k

[28] Sharon E. , Golub E., Niazov-Elkan A., Balogh D., Willner I. Analysis of telomerase by the telomeric hemin/G-quadruplex-controlled aggregation of Au nanoparticles in the presence of cysteine. Anal. Chem. 2014; 86:3153–3158. 24502233 10.1021/ac5000152

[29] Mergny J.-L. , Sen D. DNA quadruple helices in nanotechnology. Chem. Rev. 2019; 119:6290–6325. 30605316 10.1021/acs.chemrev.8b00629

[30] Wei C. , Jia G., Zhou J., Han G., Li C. Evidence for the binding mode of porphyrins to G-quadruplex DNA. Phys. Chem. Chem. Phys. 2009; 11:4025–4032. 19440632 10.1039/b901027k

[31] Ghahremani Nasab M. , Hassani L., Mohammadi Nejad S., Norouzi D. Interaction of hemin with quadruplex DNA. J. Biol. Phys. 2017; 43:5–14. 27752804 10.1007/s10867-016-9430-7 PMC5323342

[32] Lane A.N. , Chaires J.B., Gray R.D., Trent J.O. Stability and kinetics of G-quadruplex structures. Nucleic Acids Res. 2008; 36:5482–5515. 18718931 10.1093/nar/gkn517 PMC2553573

[33] Ai T. , Yang Q., Lv Y., Huang Y., Li Y., Geng J., Xiao D., Zhou C. Insight into how telomeric G-quadruplexes enhance the peroxidase activity of cellular hemin. Chem. - Asian J. 2018; 13:1805–1810. 10.1002/asia.201800464 29718585

[34] Kremer M.L. Decomposition of hydrogen peroxide by haemin. Dependence of reaction velocity on pH. Trans. Faraday Soc. 1965; 61:1453–1458.

[35] Dolinnaya N.G. , Ogloblina A.M., Yakubovskaya M.G. Structure, properties, and biological relevance of the DNA and RNA G-quadruplexes: overview 50 years after their discovery. Biochemistry-Moscow. 2016; 81:1602–1649. 28260487 10.1134/S0006297916130034 PMC7087716

[36] Fujii T. , Podbevšek P., Plavec J., Sugimoto N. Effects of metal ions and cosolutes on G-quadruplex topology. J. Inorg. Biochem. 2017; 166:190–198. 27665315 10.1016/j.jinorgbio.2016.09.001

[37] Li J. , Yuan T., Yang T., Xu L., Zhang L., Huang L., Cheng W., Ding S. DNA-grafted hemin with preferable catalytic properties than G-quadruplex/hemin for fluorescent miRNA biosensing. Sens. Actuators, B. 2018; 271:239–246.

[38] Li W. , Li Y., Liu Z., Lin B., Yi H., Xu F., Nie Z., Yao S. Insight into G-quadruplex-hemin DNAzyme/RNAzyme: adjacent adenine as the intramolecular species for remarkable enhancement of enzymatic activity. Nucleic. Acids. Res. 2016; 44:7373–7384. 27422869 10.1093/nar/gkw634 PMC5009756

[39] Liang W. , Carraro F., Solomon M.B., Bell S.G., Amenitsch H., Sumby C.J., White N.G., Falcaro P., Doonan C.J. Enzyme encapsulation in a porous hydrogen-bonded organic framework. J. Am. Chem. Soc. 2019; 141:14298–14305. 31426638 10.1021/jacs.9b06589

[40] Chen J. , Zhang Y., Cheng M., Guo Y., Sponer J., Monchaud D., Mergny J.-L., Ju H., Zhou J. How proximal nucleobases regulate the catalytic activity of G-quadruplex/hemin DNAzymes. ACS Catal. 2018; 8:11352–11361.

[41] Li J. , Wu H., Yan Y., Yuan T., Shu Y., Gao X., Zhang L., Li S., Ding S., Cheng W. Zippered G-quadruplex/hemin DNAzyme: exceptional catalyst for universal bioanalytical applications. Nucleic. Acids. Res. 2021; 49:13031–13044. 34878146 10.1093/nar/gkab1178 PMC8682752

[42] Shumayrikh N.M. , Warren J.J., Bennet A.J., Sen D. A Heme•DNAzyme activated by hydrogen peroxide catalytically oxidizes thioethers by direct oxygen atom transfer rather than by a compound I-like intermediate. Nucleic. Acids. Res. 2021; 49:1803–1815. 33476369 10.1093/nar/gkab007 PMC7913675

[43] Kejnovská I. , Renčiuk D., Palacký J., Vorlíčková M. Yang D. , Lin C. CD study of the G-quadruplex conformation. 2019; 2035:NY Humana 25–44.G-Quadruplex Nucleic Acids: Methods and Protocols. 10.1007/978-1-4939-9666-7_2 31444742

[44] del Villar-Guerra R. , Trent J.O., Chaires J.B. G-Quadruplex secondary structure obtained from circular dichroism spectroscopy. Angew. Chem., Int. Ed. 2018; 57:7171–7175. 10.1002/anie.201709184 PMC5920796 29076232

[45] Largy E. , Marchand A., Amrane S., Gabelica V., Mergny J.L. Quadruplex turncoats: cation-dependent folding and stability of quadruplex-DNA double switches. J. Am. Chem. Soc. 2016; 138:2780–2792. 26837276 10.1021/jacs.5b13130

[46] Ha S.H. , Ferrell J.E. Thresholds and ultrasensitivity from negative cooperativity. Science. 2016; 352:990–993. 27174675 10.1126/science.aad5937 PMC5184821

[47] Adegbenro A. , Coleman S., Nesterova I.V. Stoichiometric approach to quantitative analysis of biomolecules: the case of nucleic acids. Anal. Bioanal. Chem. 2022; 414:1587–1594. 34800148 10.1007/s00216-021-03781-y PMC8766926

[48] Debnath M. , Farace J.M., Johnson K.D., Nesterova I.V. Quantitation without calibration: response profile as an indicator of target amount. Anal. Chem. 2018; 90:7800–7803. 29916241 10.1021/acs.analchem.8b02053

[49] Zhu J. , Zhang L., Dong S., Wang E. How to split a G-quadruplex for DNA detection: new insight into the formation of DNA split G-quadruplex. Chem. Sci. 2015; 6:4822–4827. 29142717 10.1039/c5sc01287b PMC5667574

[50] Connelly R.P. , Verduzco C., Farnell S., Yishay T., Gerasimova Y.V. Toward a rational approach to design split G-quadruplex probes. ACS Chem. Biol. 2019; 14:2701–2712. 31599573 10.1021/acschembio.9b00634 PMC7179085

[51] Zhang K. , Wang K., Zhu X., Gao Y., Xie M.H. Rational design of signal-on biosensors by using photoinduced electron transfer between Ag nanoclusters and split G-quadruplex halves-hemin complexes. Chem. Commun. 2014; 50:14221–14224. 10.1039/c4cc06664b 25284278

[52] He L. , Chen F.M., Zhang D.L., Xie S.T., Xu S.J., Wang Z.M., Zhang L.L., Cui C., Liu Y.L., Tan W.H. Transducing complex biomolecular interactions by temperature-output artificial DNA signaling networks. J. Am. Chem. Soc. 2020; 142:14234–14239. 32677826 10.1021/jacs.0c05453

[53] Kelley S.O. What are clinically relevant levels of cellular and biomolecular analytes?. ACS Sensors. 2017; 2:193–197. 28723142 10.1021/acssensors.6b00691

[54] Zhao Y. , Chen F., Li Q., Wang L., Fan C. Isothermal amplification of nucleic acids. Chem. Rev. 2015; 115:12491–12545. 26551336 10.1021/acs.chemrev.5b00428

[55] Lake R.J. , Yang Z.L., Zhang J.L., Lu Y. DNAzymes as activity-based sensors for metal ions: recent applications, demonstrated advantages, current challenges, and future directions. Acc. Chem. Res. 2019; 52:3275–3286. 31721559 10.1021/acs.accounts.9b00419 PMC7103667

[56] Du Y. , Dong S. Nucleic acid biosensors: recent advances and perspectives. Anal. Chem. 2017; 89:189–215. 28105831 10.1021/acs.analchem.6b04190

[57] Ade C. , Brodszkij E., Thingholm B., Gal N., Itel F., Taipaleenmäki E., Hviid M.J., Schattling P.S., Städler B. Small organic catalase mimic encapsulated in micellar artificial organelles as reactive oxygen species scavengers. ACS Appl. Polym. Mater. 2019; 1:1532–1539.

