# Supplementary material for: Activatable G-quadruplex based catalases for signal transduction in biosensing
Source: Nucleic Acids Res. 2023 Feb 2;51(4):1600–7. doi: 10.1093/nar/gkad031 (PMC9976883; doi:10.1093/nar/gkad031)
Supplement: gkad031_Supplemental_File [file gkad031_supplemental_file.pdf]

Activatable G-quadruplex based catalases for signal transduction in biosensing

Elzbieta E. Iwaniuk[1], Thuwebat Adebayo[1], Seth Coleman[1], Caitlin G. Villaros[1], Irina V. Nestrova[1],*

[1] Department of Chemistry and Biochemistry, Northern Illinois University, DeKalb, IL 60115, USA
* To whom correspondence should be addressed. Tel: 1-815-753-6843; Email: inesterova@niu.edu

SUPPLEMENTARY INFORMATION

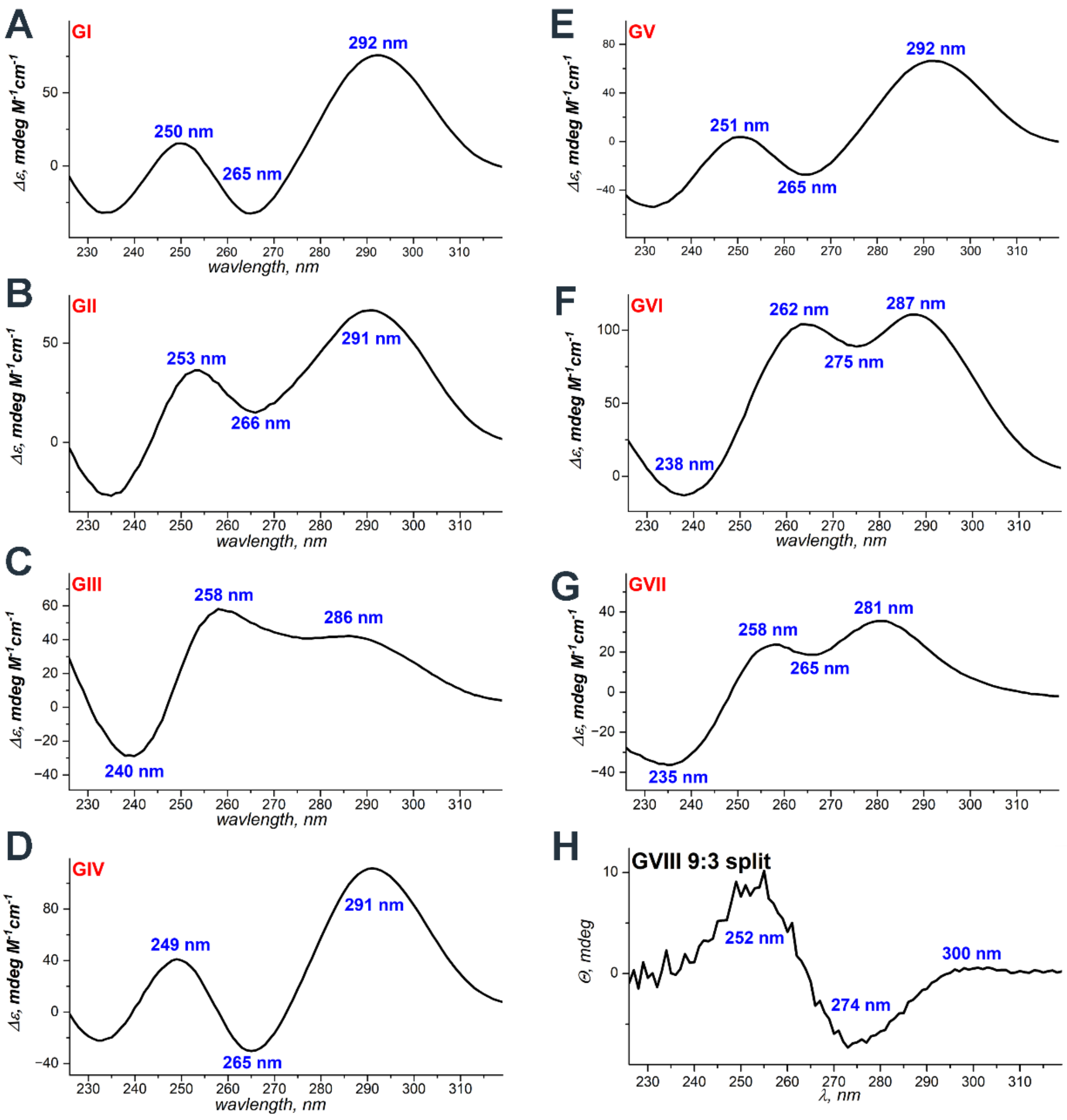

Figure S1. CD spectra of full quadruplexes GI – GVII (A – G) and the quadruplex system GVIII 9 : 3 split (H). All oligonucleotide sequences are provided in Table 1 (main text). The measurements were performed in PBS buffer (pH 7.5); oligonucleotide concentrations were ~ 5 μM.

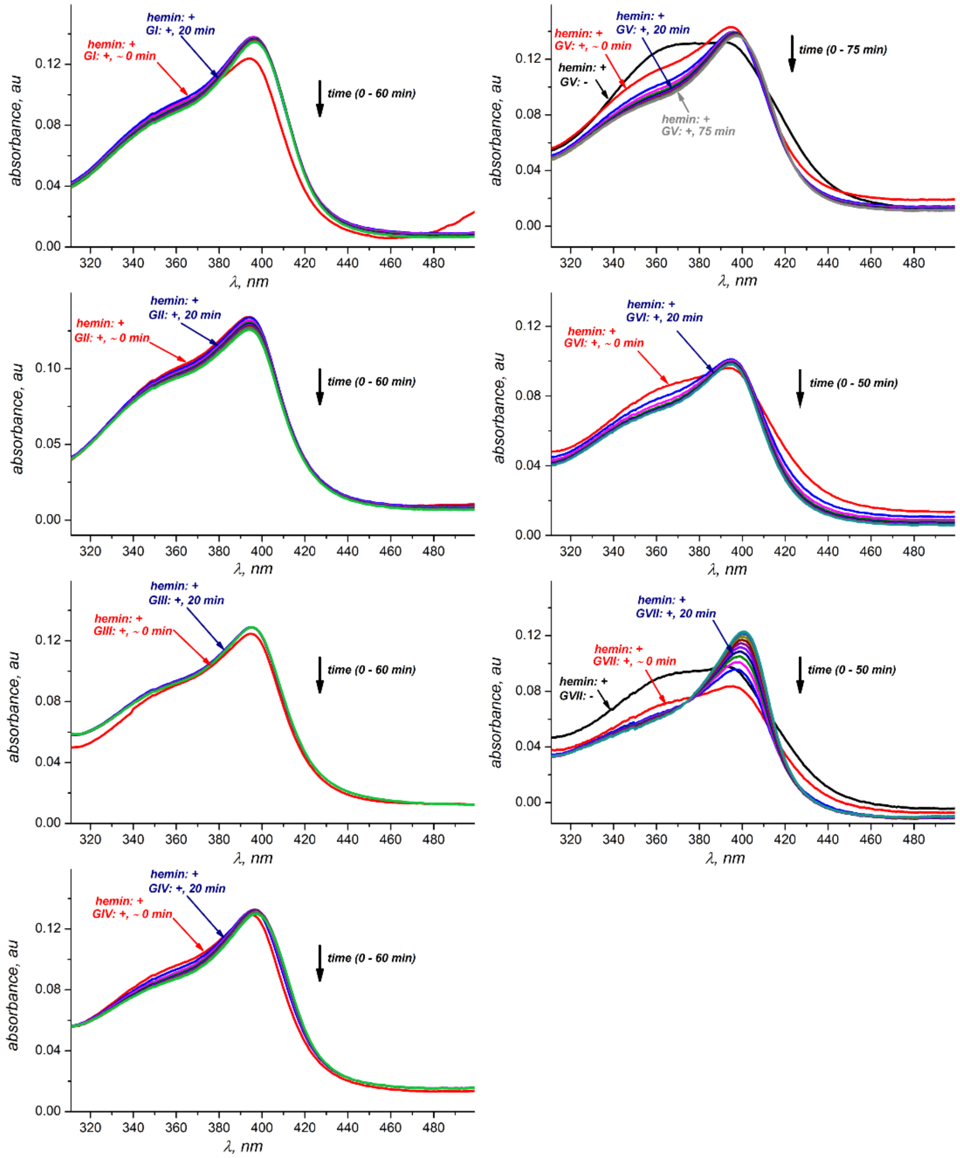

Figure S2. Bathochromic shift and increase in intensity of hemin's Soret band indicates hemin's de-aggregation upon interacting with GI – GIV and GVI – GVII. Similar trends are observed for quadruplex GV (Figure 1 in the main text). Hemin concentration is 1 μM, G-quadruplex concentrations are 500 nM in PBS buffer at pH 7.50.

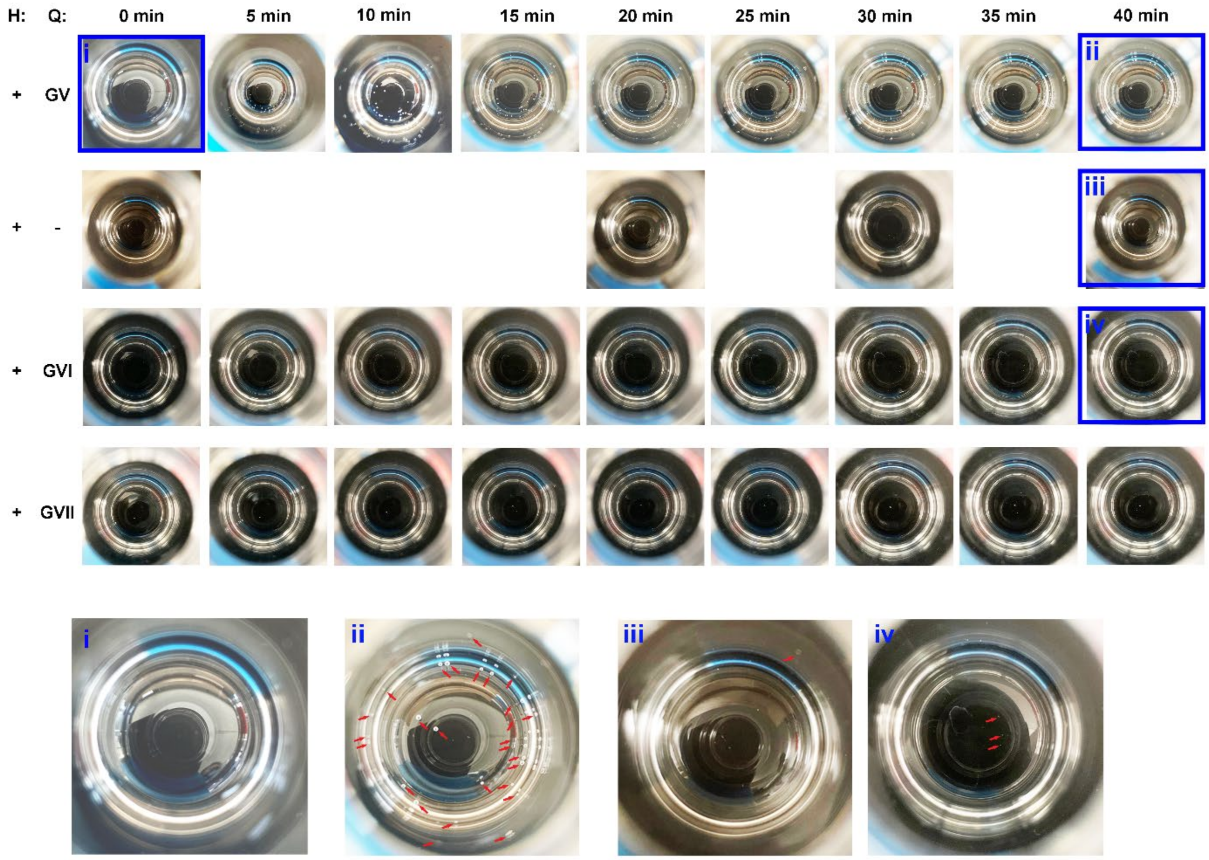

Figure S3. Top view on 20-mL scintillation vials with catalase reaction activated by quadruplexes GV (top row), GVI (third row), GVII (forth row), control (second row) and zoomed in frames i – iv (bottom row) as labeled. The reaction was performed in PBS buffer at pH 7.5 in presence of 1 μM hemin and 29.3 % H_2O_2. Oligonucleotides GV, GVI, and GVII were at 200 nM.

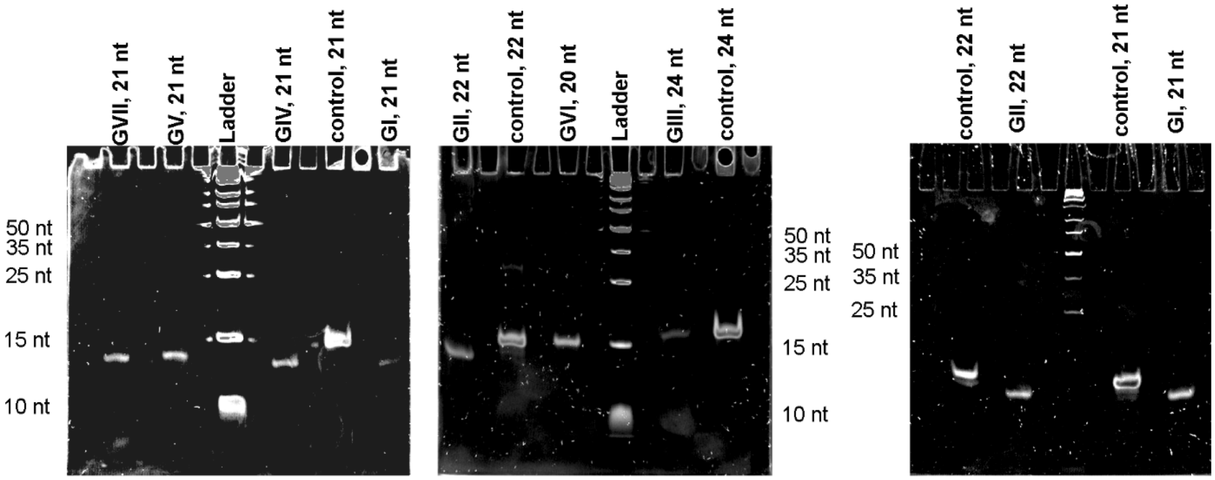

Figure S4. 20% PAGE electrophoresis in 0.5 × TBE indicates that quadruplexes GI, GII, GIV, GV, and GVII are folded in PBS pH 7.5 buffer. Quandruplexes GIII and GVI migrate as unfolded strands. Injection volume for samples of ∼4 µM oligonucleotide solutions in PBS with ~ 17 % of glycerol was 4 µL. The gels were stained in SYBR Gold (3 µL dye in 200 mL of 0.5 × TBE buffer). All the sequences are included in Table S1.

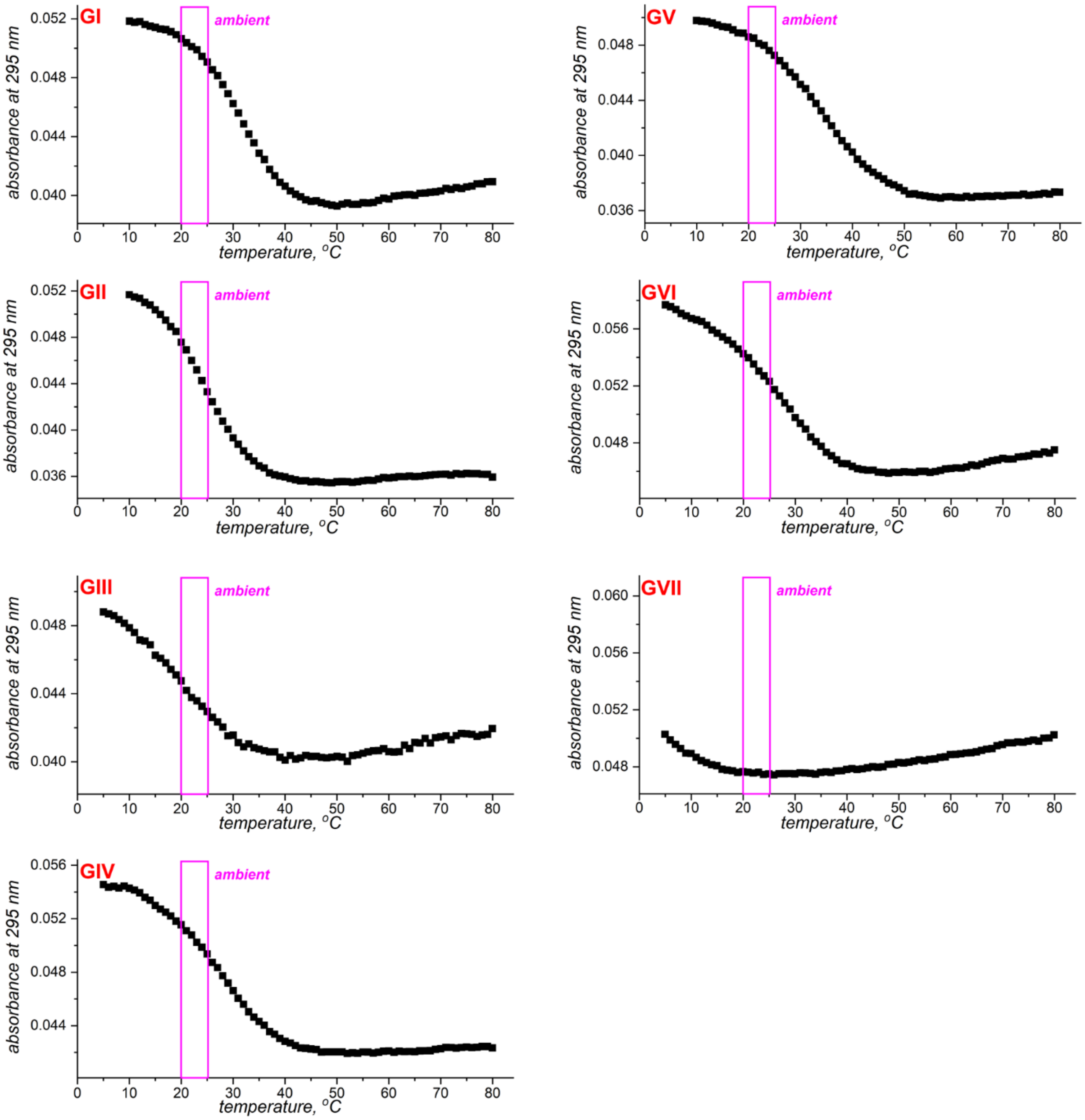

Figure S5. Thermal folding profiles indicate that quadruplexes GI – GVI are folded at room temperature (20 – 25 ºC). Quadruplex GVII does not show folding state at the evaluated temperature range. The profiles were acquired for 500 nM oligos in PBS buffer at pH 7.50. The oligonucleotides were denatured at 80 ºC for 30 minutes before cooling down at 0.2 ºC/min rate. Absorbance at 295 indicative of quadruplex formation was measured every 1 ºC. Magenta boxes indicate the range of ambient temperatures (20 – 25 ºC).

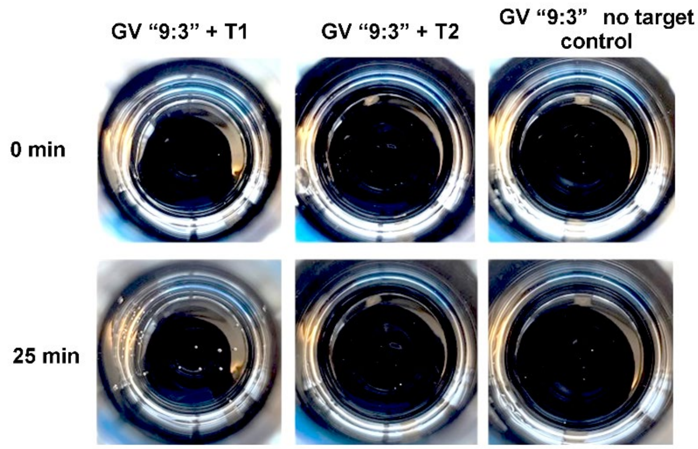

Figure S6. Representative images of GV "9:3" split response to the target T1 (left) and T2 (middle) against a control (no target, right) taken with a cell phone camera. The samples were prepared in 20-mL scintillation vials. The total sample volume was 2000 µL. Each sample in PBS buffer (pH 7.50) consisted of two quadruplex "arms" (R and L) and target (all at 500 nM concentrations), hemin at 1µM, and hydrogen peroxide at 29.3 %. Images at the upper raw ("0 min) are taken directly before target addition; images at the bottom raw ("25 min") are taken 25 min after the target addition. A large number of bubbles (especially on the left side, partially obscured by the vial neck) are observed upon addition of Target T1.

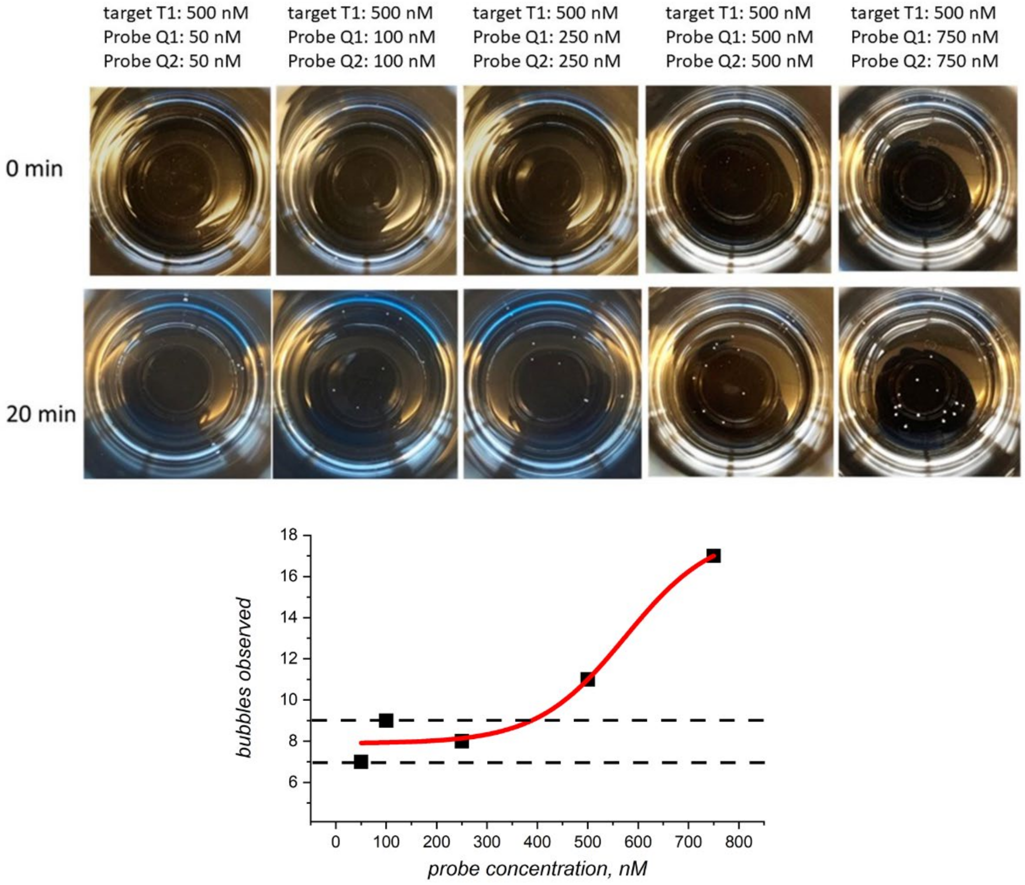

Figure S7. Stoichiometric approach reported earlier (ref. 48) is applied towards quantitative analysis of target T1. Probes Q1 and Q2 were designed to accommodate the binding model requirements discussed in ref. 48. Sharp increase in amount of bubbles (so called "inflection point", bottom raw, images for probes at 500 nM and 750 nM) indicates to the target amount. The position of the "inflection point" is determined using "Method 1" from ref 48 (main text) to be at 400 nM (for target present at 500 nM level). Samples were prepared as described for split quadruplexes. Further probe design refinements to enable lower "background" (bubble formation in 50 nM, 100 nM, and 200 nM samples) is currently under way. The refinements are expected to improve accuracy.

Table S1. Sequences of oligonucleotides used throughout the studies.

**Name**	**Sequence**
Full G-quadruplexes:	
GI	5'- GGG TTA GGG TTA GGG TTA GGG-3'
GII	5'- A GGG TTA GGG TTA GGG TTA GGG-3'
GIII	5'-ATT GGG TTA GGG TTA GGG TTA GGG-3'
GIV	5'- GGG TTA GGG TGT GGG TTA GGG-3'
GV	5'- GGG TTA GGG TCT GGG TTA GGG-3'
GVI	5'- A GG TTTT GG CA GGG TTTT GG T-3'
GVII	5'- A GGG TT GGG TGT GGG TT GG-3'
Split G-quadruplexes and targets. Domains "a" are underlined; domains "b" are double-underlined, domains "c" are wave-underlined, domains "d" are dash-underlined, domains "c'" are *italicized and wave-underlined*, domains "d'" are *italicized and dash-underlined*.	
Target T1	5'-*TATTTCTTATTCATATTCAGGAAGACATCGAGACCAGGGCACGC*-3'
Target T2	5'-*TATTTCTTATTCATATTCAGGAAGA*TTTTTTTT*CATCGAGACCAGGGCACGC*-3'
GI 6 : 6 L	5' TTAGGGTTAGGG*TCTTCCTGAATATGAATAAGAAATA*-3'
GV 6 : 6 L	5' TCTGGGTTAGGG*TCTTCCTGAATATGAATAAGAAATA*-3'
GVIII 6 : 6 L	5'-ATTGGGATTGGG*TCTTCCTGAATATGAATAAGAAATA*-3'
GIX 6 : 6 L	5'-TCTGGGATTGGG*TCTTCCTGAATATGAATAAGAAATA*-3'
GI 6 : 6 R<br>GV 6 : 6 R<br>GVIII 6 : 6 R<br>GIX 6 : 6 R	5'-*GCGTGCCCTGGTCTCGATG*GGGTTAGGG-3'
GI 9 : 3 L	5' GGGTTAGGGTTAGGG*TCTTCCTGAATATGAATAAGAAATA*-3'
GV 9 : 3 L	5' GGGTCTGGGTTAGGG*TCTTCCTGAATATGAATAAGAAATA*-3'
GVIII 9 : 3 L	5'-AGGGATTGGGATTGGG*TCTTCCTGAATATGAATAAGAAATA*-3'
GIX 9 : 3 L	5'-AGGGTCTGGGATTGGG*TCTTCCTGAATATGAATAAGAAATA*-3'
GI 9 : 3 R<br>GV 9 : 3 R	5' *GCGTGCCCTGGTCTCGATG*GGGTTA-3'
GVIII 9 : 3 R<br>GIX 9 : 3 R	5'-*GCGTGCCCTGGTCTCGATG*GGGTT-3'

Table S1 (continued)

Name	Sequence
GVIII 9 : 3 split T control L	5’-ATTTATTTTTATTTTT*TCTTCCTGAATATGAATAAGAAATA*-3'
GIX 9 : 3 split T control L	5’-ATTTTCTTTTATTTTT*TCTTCCTGAATATGAATAAGAAATA*-3'
9 : 3 split T control R	5’-*GCGTGCCCTGGTCTCGATG*TTTTT-3'
Single stranded controls for polyacrylamide gel electrophoresis	
21-nt single stranded control for PAGE	5’ – TAT TTC TTA TTC ATA TTC AGG – 3’
22-nt single stranded control for PAGE	5’ – TAT TTC TTA TTC ATA TTC AGG A – 3’
24-bp single stranded control for PAGE	5’ – TAT TTC TTA TTC ATA TTC AGG ATT – 3’
Probes for quantitative analysis (Figure S7) *(target binding domains are underlined, overlap regions are* ***bold****, G-quadruplex forming sequences are double-underlined)*	
Q1	5’ – GCGTGCCCTGGTCTCG**ATGTCT**GGGGTTA – 3’
Q2	5’ – GGGTTAGGGTTAGGG**ATGTCT**TCCTGAATATGAATAAGAAATA – 3’
